# Urban Planning and Smart City Decision Management Empowered by Real-Time Data Processing Using Big Data Analytics

**DOI:** 10.3390/s18092994

**Published:** 2018-09-07

**Authors:** Bhagya Nathali Silva, Murad Khan, Changsu Jung, Jihun Seo, Diyan Muhammad, Jihun Han, Yongtak Yoon, Kijun Han

**Affiliations:** 1 School of Computer Science and Engineering, Kyungpook National University, Daegu 41566, Korea; nathalis@netopia.knu.ac.kr (B.N.S.); jungchangsu@gmail.com (C.J.); jhseo87@netopia.knu.ac.kr (J.S.); m.diyan@netopia.knu.ac.kr (D.M.); jhhan@netopia.knu.ac.kr (J.H.); ytyoon@netopia.knu.ac.kr (Y.Y.); 2 Department of Computer Science, Sarhad University of Science and information Technology, Peshawar 25000, Pakistan; murad.csit@suit.edu.pk

**Keywords:** Big Data analytics, Big Data smart city, Big Data urban planning, smart city planning

## Abstract

The Internet of Things (IoT), inspired by the tremendous growth of connected heterogeneous devices, has pioneered the notion of smart city. Various components, i.e., smart transportation, smart community, smart healthcare, smart grid, etc. which are integrated within smart city architecture aims to enrich the quality of life (QoL) of urban citizens. However, real-time processing requirements and exponential data growth withhold smart city realization. Therefore, herein we propose a Big Data analytics (BDA)-embedded experimental architecture for smart cities. Two major aspects are served by the BDA-embedded smart city. Firstly, it facilitates exploitation of urban Big Data (UBD) in planning, designing, and maintaining smart cities. Secondly, it occupies BDA to manage and process voluminous UBD to enhance the quality of urban services. Three tiers of the proposed architecture are liable for data aggregation, real-time data management, and service provisioning. Moreover, offline and online data processing tasks are further expedited by integrating data normalizing and data filtering techniques to the proposed work. By analyzing authenticated datasets, we obtained the threshold values required for urban planning and city operation management. Performance metrics in terms of online and offline data processing for the proposed dual-node Hadoop cluster is obtained using aforementioned authentic datasets. Throughput and processing time analysis performed with regard to existing works guarantee the performance superiority of the proposed work. Hence, we can claim the applicability and reliability of implementing proposed BDA-embedded smart city architecture in the real world.

## 1. Introduction

Conventional networks have transformed into the Internet of Things (IoT), with the “connected objects” notions influenced by exponential growth in the number of smart devices [1,2]. In general, the IoT is defined as a collection of heterogeneous things that are uniquely addressable and capable of collecting and sharing information with nominal human interaction [1,3,4,5]. The notion of IoT has evolved rapidly, owing to remarkable attention received from experts and unceasing improvements in embedded technology. IoT applications vary across myriads of fields and contexts, i.e., smart city, smart homes, smart warehouse, smart agriculture, and smart healthcare [3,6,7,8,9,10,11,12].

**Figure 1:**
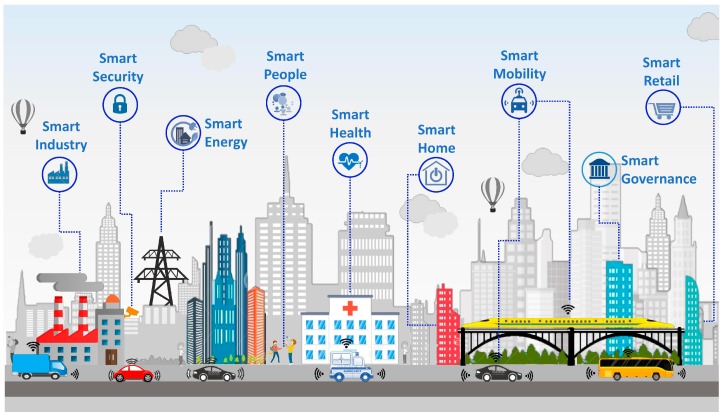
Overview of a generic smart city environment.

Novel applications have been introduced and promoted in metro urban areas to ensure quality service provision throughout the community, despite extreme urbanization and population growth. Telecity, smart city, and information city concepts were coined as a result of using information and communication technology (ICT) in municipal service provision. Siembab defines telecity as a city that uses information technology for mobility, economy, and other public services. Moreover, in a telecity every resident can access a variety of IT services on demand or on a first-come first-serve basis. Telecities comprise a mix of houses that accommodate simple and full-featured tasks incorporating a combination of old and new technologies [13]. Smart city is a concept that encapsulates all other concepts into a single architecture [14,15]. This abstract nature has popularized the smart city to stand out among others. The overview of a typical smart city is illustrated in Figure 1. In the beginning, the utmost goal of smart cities was to improve the quality of life (QoL) of urban citizens [12,16]. Experts have performed numerous studies and research on smart city designing and real-world implementation. However, a majority of the works aims to improve or incorporate one or some essential components of a smart city, i.e., parking management, waste disposal and recycling, infrastructure management, etc. Nevertheless, the efficacy of the city service provision is negatively influenced by lack of integrity. Hence, the modern urbanized world urges us to address the compelling demands for complete, flexible, and efficient smart city architectures. Accordingly, in [17], the authors have proposed a layered architecture for smart cities considering the dataflow of the urban network. In fact, this architecture comprises key components of a smart city, even though the performance is not analyzed quantitatively. A conceptual smart city architecture consists of data collection, data processing, and query handling layers has been proposed in [18] to facilitate seamless and ubiquitous communication among connected heterogeneous devices.

A city’s service provision efficiency is highly correlated with data collection followed by data manipulation and decision generation. Expansion of wireless sensor networks (WSN) increase the amount of urban data circulating across the network, which generates urban Big Data (UBD). In fact, the efficacy of smart city services is empowered by real-time processing of UBD. Conventional data processing mechanisms are unable to meet real-time processing demands with accelerating data growth. Conventional data processing mechanisms use statistical methods to analyze large datasets, in order to extract useful hidden data [19]. Predictive analytics is a commonly used conventional data analyzing mechanism that is based on statistical methods. Predictive analytics can be further categorized with respect to the outcome variable [20]. However, the direct application of predictive analytics has been questioned by the domain experts due to statistical insignificance in large datasets, challenges in efficient computing, and distinctive Big Data (BD) characteristics, i.e., heterogeneity, noise accumulation, false positive correlation, and incidental endogeneity [21].

Hence, experts are keen to work on highly promising smart city solutions that embed Big Data analytics (BDA). Furthermore, accurate and efficient data processing abilities offered with BDA assures intelligent decision management in modern smart cities [22,23] such that embedding BDA to smart cities improves quality of urban services. Subsequent to increased quality of service (QoS), BDA-embedded smart city increases the QoL of its urban population [24,25]. A smart city architecture embedded with BDA to handle enormous data processing and analyzing demands has been proposed by Jara et al. [26]. In [26], traffic BD of SmartSantander city have been analyzed by the authors. Another work on SmartSantander testbed has evaluated the benefits of embedding BDA operations [22]. Here, the authors tested historic data as well as real-time data on the proposed BD platform on the SmartSantander testbed. Since these approaches were evaluated only for the performance improvement of certain components, a holistic city architecture that collects and processes data to derive intelligent decisions for the performance betterment of the smart city is still a compelling demand.

Herein, we propose a BDA-embedded smart city architecture that ensures reliable and efficient data management to derive real-time intelligent decisions. The proposed three-layered architecture consolidates data aggregation, data manipulation, and service management tasks. The data management component is considered to be the brain of the architecture as it performs data filtration, analysis, processing, and storing of valuable data. Filtration mechanisms are introduced to the architecture, to further expedite processing and analysis. MapReduce on Hadoop is used for offline data processing and Apache Spark is used for online data processing. Intelligent agents and brokers of the architecture generate and transfer intelligent decisions to the service management layer, to formulate desired urban services. Experimentation results revealed that the occupied filtration technique has significantly improved the processing performance in terms of processing time and data throughput. Moreover, performance superiority of selected processing mechanisms, namely Apache Spark for online streaming data processing and Hadoop MapReduce for offline batch processing, has been proven by the results. Furthermore, we compared processing time and throughput performance of the proposed work with two previously reported BD analytical systems tested on smart city environments. Owing to performance improvement gained through integrated normalizing and filtering techniques, we could claim that the proposed work has contributed to the existing knowledge base.

The remaining sections of the paper are formulated as follows. Some recent studies that have been performed on smart cities and BD are presented in Section 2. Section 3 elaborately discusses the proposed architecture for BDA-embedded smart city. Performance analysis and discussion are presented in Section 4. Finally, Section 5 outlines the conclusions of the work.

## 2. Related Works

Experts in both industry and academia have derived various solution approaches to optimize urban service provision incorporating the benefits of IoT-based smart cities. Accordingly, numerous experimental studies and testbed-based studies were performed to identify the challenges of smart cities and to determine the solutions for identified challenges. An experimental solution that evaluates testbed city services has been proposed in [27]. In this work, a large-scale IoT infrastructure has been deployed in Santander city. The beauty of this work is that it has been developed in a user-friendly manner to enable the tester to evaluate smart city services in different environments. Consequently, this experimental setting assists in planning non-identical smart cities. Moreover, the testbed experimentation results claimed that the architecture proposed in [27] has derived solutions to many challenges in current literature. Nevertheless, data gathered from deployed sensors, which are essential in real-world smart city deployment were not evaluated in this work. A computational architecture aiming to improve the traceability of cities has been proposed in [28], incorporating radio frequency identification (RFID) sensing. This architecture reliably and transparently acquires user information from a heterogeneous environment. In this work, authors incorporated citizens’ mobility patterns, to minimize deviation from actual movements during transportation route management. Moreover, many literature reports have focused on cloud-based smart city development, owing to invaluable benefits facilitated through cloud [29,30,31]. SmartCityWare is a cloud-based service-oriented middleware platform that assists smart city development and municipal service provision [32]. SmartCityWare provides a virtual environment to implement smart city applications. This cloud-based work perceives every function as a service of a smart city. The key functionality of SmartCityWare is to enable seamless integration of components and smooth operation among all services. To attain this facility, the services are distributed among various clouds, devices, and fogs.

BDA tools depend on the core characteristics of BD, known as VVV (volume, variety, and velocity). A majority of BDA-embedded smart city works have incorporated offline BD processing for urban management and planning, since real-time BD processing and intelligent decision-making are challenging and tedious. Data collected from a million smart meters within a year is calculated and presented in Table 1, assuming that a record size is 5 KB [33]. As shown in Table 1, the set of smart meters collect 2920 terabytes (TB) for a period of one year with a frequency of record per every 15 min. Considering the data growth shown in the example, we can claim the necessity for real-time data processing techniques in smart cities. Smart environments of the modern era tend to rely on more sophisticated data management techniques, since conventional data processing techniques are not capable of catering for the exponential data growth.

The QoL of urban citizens is improved by deriving autonomous intelligent decisions from data generated within the urban network. To assist data creation and data sharing, a large number of sensors and heterogeneous devices are deployed within the smart city environment. In fact, real-time BD processing assists tremendously in designing and managing the cities, while transforming them in to smart cities. Moreover, BDA ensures reliability of sensitive and critical decisions, which on the other hand improve the efficacy and revenue of municipal authorities. City data and analytic platform (CiDAP), which has been developed on Hadoop, aims to improve service design and use [22]. Results from archive data processing and real-time processing are exposed to various applications via CiDAP. The architecture is tested with simple queries and complex queries to evaluate its performance in terms of throughput. The results claimed that throughput of urban network has been improved by the CiDAP architecture. SCOPE [34] and FIWARE [35] are two commercially proposed architectures that aim on maximizing the benefits of BDA embedding. SCOPE is a smart city cloud-based open platform ecosystem designed and developed by Boston University. FIWARE is a framework that enables intelligent application development in the future internet. A four-tier smart city architecture that analyzes UBD has been proposed in [36]. The architecture consists of number of heterogeneous sensors and relay nodes. Another four-layered architecture based on fog computing has been proposed in [37]. This architecture periodically monitors public infrastructure to identify infrastructure changes over the time. This architecture employs a group of low-cost, non-invasive, and reliable sensors to collect BD at the bottom layer. IBM [38] and AGT [39] have developed few other data platforms for smart cities. The key drawback of these projects is inability to generalize and that they are not openly available for different contexts. Despite the remarkable attention and works conducted around BDA-embedded smart cities, actual implementation is still challenging in many ways.

## 3. Proposed Scheme

The proposed smart city framework that embeds BDA comprises of three tiers, namely, data aggregation, data management, and service management. The following subsections discuss the overview and functionalities of each layer of the proposed smart city architecture.

### 3.1. Overview of the Proposed Architecture

The dataflow and workflow of the proposed architecture is bottom-up in design, starting with data creation and collection level, data management level, and service management level. Smart things, vehicles, sensors, devices, and actuators build the bottom line of the city architecture. The bottom line creates data and shares them among others that are connected within the network. Various communication technologies that support short-range communication and long-range communication facilitate data collection and sharing. Aggregated data are transferred to the middle layer to determine valuable data from gathered raw data with the aid of data filtration and data analysis. Intelligent decision agents classify valuable data and derive intelligent decisions. Since decisions are the key factors for performance improvement the proposed scheme aims to derive intelligent decisions from offline data processing as well as online data processing. The service management tier is responsible for formulating and generating smart city service events with respect to derived intelligent decisions.

**Figure 2:**
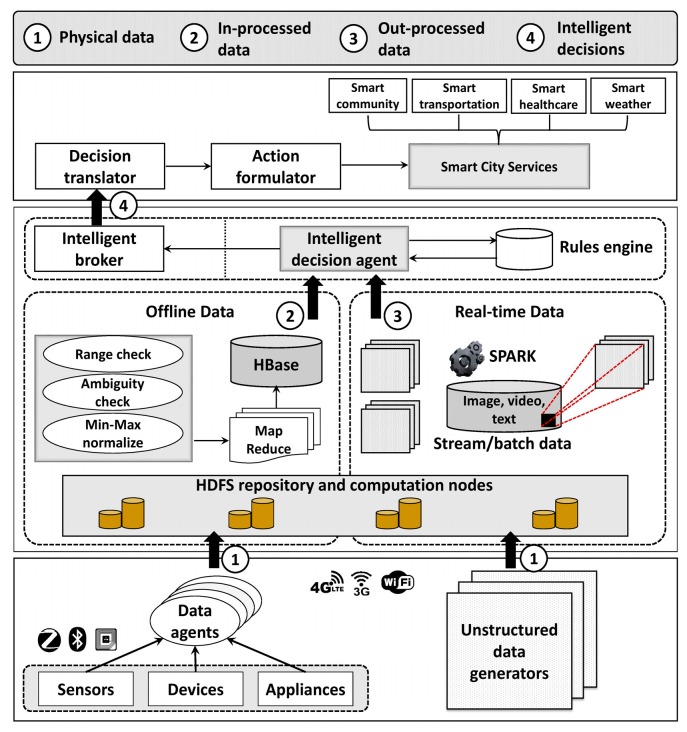
Dataflow and workflow of the proposed architecture.

The utmost goal of the proposed work is to improve data processing and decision-making performance of a realistic smart city architecture by embedding data filtration techniques and BDA. In the recent past, experts have actively experimented the benefits of embedding BDA into smart city domains. To improve BD processing in smart city environments, the proposed work integrates min-max normalization and data filtration techniques i.e., range checking and ambiguity checking to reduce the amount of noisy, corrupted, and ambiguous data. The normalization process determines threshold limits for minimum desired value $K_{\min}$ and maximum desired value $K_{\max}$ in order to distinguish potentially valuable data. These $K_{\min}$ and $K_{\max}$ values were obtained by initial batch processing of stated authentic datasets. Furthermore, normalized and filtered data reduce the unnecessary redundant load on the Hadoop processing cluster. Intelligent decision agents and intelligent brokers derive precise decisions in real time, using processed data and the rules engine. Incorporated filtration mechanisms reduce noisy or corrupted data from the dataset, thus reducing unnecessary load on the Hadoop cluster. Moreover, the proposed architecture occupies a dual-node Hadoop cluster instead of a single node Hadoop to further expedite the processing tasks. Figure 2 presents the overview structure of the proposed smart city.

### 3.2. Data Aggregation Layer

Actual implementation of smart city encompasses simple and complex application contexts, exhaustive computational processes, and BD. In fact, computations and data are the two most important distinctive features for smart city realization [17]. As stated before, the smart city aims to enhance the QoS of urban services, such as waste management, healthcare, intelligent transportation, etc. The betterment of QoS relies upon comprehensive city data collection process, which gathers data related to every single city service task. Consequent to accelerated data generation by connected things and people, data collection has become a tedious and highly challenging task. Owing to cost and energy-efficient operations, sensors are widely used for city data collection purposes. Thus, the bottom layer of the smart city architecture deploys piles of sensors to cover the entire city. Extensive sensor deployment assures timely data acquisition, leading to a smarter city [27]. Data from environment and things are gathered by the deployed sensor network. It is worth noting that data and data types acquired by sensors vary with the implanted context, i.e., mobile things, vehicles, domestic appliances, outdoors, indoors, etc. Data generated from sensors and other connected things can be either unstructured or semi-structured. In the proposed architecture, semi-structured data are represented by JavaScript object notation (JSON) adhering to representational state transfer (REST). Hence, the data agents based on REST aggregate both data types from sensors and other things deployed in the network. Subsequently, gathered data are transferred to the middle layer to perform processing and storing. However, an option to bypass data agents is introduced in the proposed work to mitigate bottleneck creation at data agents.

### 3.3. Data Management and Storage Layer

Data management layer is the core layer of proposed architecture as it performs main data processing tasks, namely, filtering, normalizing, analyzing, processing, and storing. The data management layer is sub-divided into offline data management and online data management. A collection of data management platforms including Hadoop Distributed File System (HDFS), HBase, MapReduce, and Spark are incorporated in this layer to facilitate the abovementioned data processing tasks. HBase is a NoSQL repository on HDFS and it stores semi-structured data gathered from the smart city data agents. The rationale for using HBase is to enhance data processing performance with aid of in-memory caching, real-time lookups, and server programming. Moreover, HBase is fault tolerant and assists in usability improvements. HBase gathers semi-structured data in JSON files. Real-time data processing is facilitated together with offline data processing at HBase. Performance degradation problems are addressed by introducing data restrictions. Data filtration processes data that is received from the data agents to refrain from erroneous, ambiguous, and redundant data. Subsequently, min-max filtration is introduced to normalize filtered data. Normalized data are analyzed using MapReduce. MapReduce analysis aims to perform offline processing on large sets of historic data. The importance of offline data processing is to determine promising changes that are required in urban planning, that lead to performance enhancements. MapReduce maps filtered data into different data sets and then associated mapped data are merged together to create smaller datasets.

The proposed work incorporates ambiguity checking to filter semi-structured data. For each parameter, corresponding rules are defined in advance to identify ambiguities. In general, heterogeneous data collection process gathers erroneous data, which may cause faulty behaviors in the smart city environment. Therefore, the proposed framework filters gathered data before normalization process, such that it avoids progression of adverse effects of outliers towards the decision-making process. Data normalization is embedded in order to maintain data reliability. UBD could be voluminous and heterogeneous with a considerable portion of erroneous, ambiguous, and redundant data. Outliers resulting from erroneous or ambiguous data affect the reliability of analyzed results that determine the intelligent decisions. To avoid any potential adverse influences, outliers will be identified by the normalization procedure and remove them from the dataset transferred for analysis. Since smart city data collection is minimally controlled, data collection is prone to missing values, ambiguous data, and out-of-range values. Normalizing is used as a data pre-processing mechanism. Min-max normalizing is a generic normalization method that normalizes all features or parameters to a defined interval, typically [0, 1] [40]. Here, each feature is standardized and transformed, such that the mean is zero and standard deviation is one. Min-max normalizing is widely used in practical applications, even though the productivity is influenced by background information. Accordingly, it prevents progression of adverse effects of outliers towards the decision-making process. In general, data reliability becomes questionable when large sets of data contain numerous variables that belong to different scales and measures. Nevertheless, the normalization process assures data reliability by scaling all data into an acceptable range. We obtained $K_{\min}$ and $K_{\max}$ values with the initial batch processing of stated authentic datasets. Hence, $K_{\min}$ and $K_{\max}$ are defined in advance for all variables in the smart city environment. Hence, we can claim that heterogeneity is not a concern in this regard, since $K_{\min}$ and $K_{\max}$ are not common values for all variables. For each data value K, the normalized value is obtained by applying the normalizing equation. Normalized value for data K is represented $K_{\mathrm{norm}}$. If $K_{\mathrm{norm}}$ is not scaled between 0 and 1, the K value is removed from the analysis dataset. Algorithm 1 presents the algorithm for min-max normalization. HBase performs both data filtration and data normalization tasks.

**Algorithm 1** Data Normalization with Min-Max Approach
***BEGIN***
  **Input:** Data value *K*, Minimum acceptable value *K*_min_, Maximum acceptable value *K*_max_  **Output:** Normalized data value *K*_norm_  1. Define normalization range (0 to 1)  2. Define boundaries for *K*_min_ and *K*_max_  3. Check if (*K*_min_ == *K*_max_ ∥ *K*_max_ < *K*_min_   ● Go back to step 2 and continue  4. For each *K*,   ● $K_{\mathrm{norm}}=\frac{K-K_{\min}}{K_{\max}-K_{\min}}$   ● If (0 ≤ *K*_norm_ ≤ 1)    Add *K* to initial dataset for analysis   ● Else    Discard *K* value
***END***


As mentioned previously, in this work, initial batch processing results and background information are used to determine $K_{\min}$ and $K_{\max}$ threshold values for each parameter, thus excluding ambiguous and out-of-range data that do not fall within defined scales [41]. The min-max normalizing technique can be expressed as below.

(1) $$K_{\mathrm{norm}}=\frac{K-K_{\min}}{K_{\max}-K_{\min}}\left(n-m\right)+m\;$$

Herein, $K_{\mathrm{norm}}$ is the normalized value of K, when $K_{\min}$ is minimum desired value, $K_{\max}$ is maximum desired value, n is the upper limit for normalizing interval (1), and m is the lower limit for normalizing interval (0). For example, assume normalizing of domestic water consumption values 58,000 and 134,000 when $K_{\min}$ is 10,000, $K_{\max}$ is 110,000, n is one, and m is zero. The normalized value for 58,000 is 0.48, which lies within the range [0, 1] and normalized value for 134,000 is 1.24, which deviates from the range [0, 1] as shown below.

$$K_{1}=\frac{58000-50000}{90000-50000}\left(1-0\right)+0=0.48\;$$

$$K_{2}=\frac{134000-50000}{90000-50000}\left(1-0\right)+0=1.24\;$$

Accordingly, min-max normalization determines potential outliers and avoids progressing of adverse influence of the outliers.

The proposed work uses HDFS as the storage medium of the smart city, owing to its scalability and distributed nature, which assists in BD processing. Since SQL queries are not permitted on Hadoop cluster, Apache Spark handles querying of large datasets on HDFS. As unstructured data are voluminous compared to semi-structured data, they are processed and analyzed extensively prior to storing in HBase. To maintain processing performance of HBase, voluminous unstructured data processing is not performed in HBase. Instead, real-time data processing and unstructured data processing are handled by Spark, which derives real-time decisions from streaming data. External processing at Spark facilitates flexibility, while increasing the processing efficiency. External Spark processing creates semi-structured data that are storable in HBase. The intelligent agents derive decisions corresponding to processed data considering rules defined in the rules engine. The rules are created from archived data processing, which are sufficiently discussed in Section 4. Further, threshold values and constraints for each dataset are defined in the rules engine. Subsequently, the intelligent broker represents derived decisions adhering to a pre-defined vocabulary shared throughout the city framework. Shared vocabulary simplifies decision translation throughout the city architecture, while avoiding ambiguities.

### 3.4. Service Management Layer

**Figure 3:**
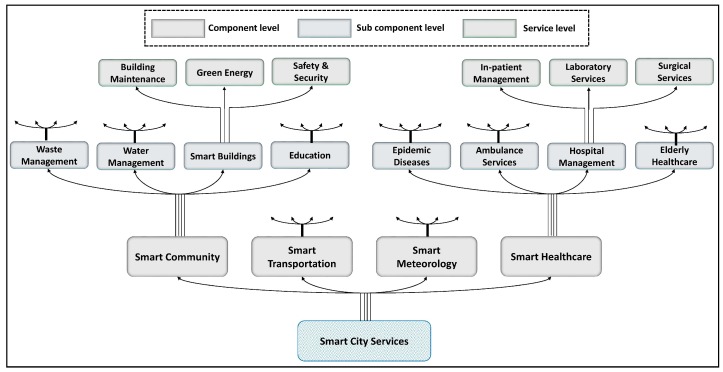
Expanded composition of service management layer.

Service management layer bridges smart city architecture with urban citizens by connecting users and city operations. The city architecture aims to enhance QoL of citizens by formulating services at the service layer according to the decisions received from intelligent brokers. Once decision information are received at the service layer, the decision data are converted into action data. Accordingly, action data are formulated to determine desired action that should be performed by smart city services components (SCSC). Figure 3 presents the hierarchical breakdown of proposed SCSC.

Figure 3 is not the exhaustive representation of all city components but shown as an example for component layering of generic SCSC. From top to bottom, the three layers of SCSC are service, sub-component, and component layers. Component level of the SCSC relates to action formulator. Since action formulator adheres with the shared vocabulary, component layer unicasts received action to the service layer through sub-component layer. Action unicasting avoids unnecessary dataflow that creates bottlenecks and congestion. Action unicasting is assured by the defined ontology.

## 4. Results and Data Analysis

In this work, the smart city architecture is designed to perform real-time data processing, which improves autonomous decision generation. Analysis of large datasets belonging to various fields influence the performance improvement of city operations. Authentic datasets mentioned in Table 2 were obtained to evaluate data processing performance. Initial datasets are not filtered and contained noisy data. Hence, data filtration and min-max normalization techniques were incorporated to improve large dataset processing performance.

### 4.1. Dataset Information

Authentic datasets for energy consumption, water consumption, road traffic, parking spaces, and pollution measurements were obtained from openly available data portals [42,43,44,45]. Smart meters of 61,263 smart homes in Surrey (Canada) have collected energy consumption and water consumption data. Sensors deployed in Aarhus city (Denmark) gathered pollution measurement data, parking lot data, and city traffic data. Pre-defined sensor pairs were used to determine road traffic in between the two points. Sensors deployed in selected parking lots of Aarhus city gathered parking information including used spaces and availability. Toxic gas concentrations, i.e., Ozone (O_3_), Nitrogen Dioxide (NO_2_), and Carbon Monoxide (CO) within city suburbs are recorded in the pollution measurements dataset. All datasets used in data processing evaluation are authenticated and openly accessible. Open government license of city of Surrey, Canada covers water and energy datasets. Traffic data, parking lot data, and pollution data are semantically annotated datasets for the CityPulse EU FP7 project and the data are licensed under Creative Commons Attribution 4.0 International License. Information corresponding to all datasets are presented in Table 2.

### 4.2. Simulation Scenario for Data Analyzing

**Figure 4:**
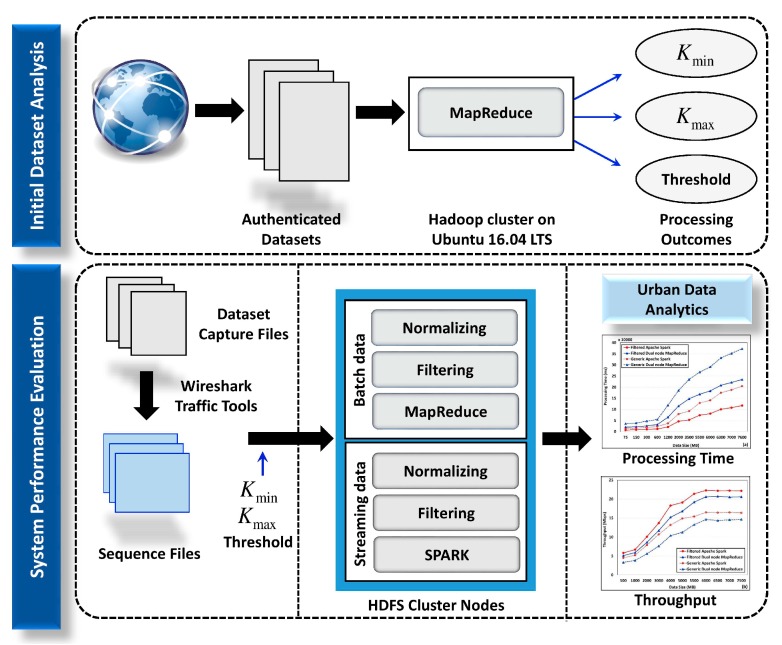
Experimental configuration scenario for initial data analysis and system evaluation.

A computer operated on Ubuntu 16.04LTS with core i5 processor and 8 GB memory is used to host the Hadoop cluster that analyzes aforementioned datasets. Real-time traffic was generated by Wireshark libraries. We used traffic generator tools of Wireshark to generate traffic from a capture file. Accordingly, generated traffic was retransmitted towards the implemented Hadoop cluster. Data traffic in pcap format were analyzed using Hadoop-pcap and Hadoop-pcap-scr-de libraries. Hadoop-pcap and Hadoop-pcap-scr-de process network packets and generate SequenceFiles. SequenceFile generation is advantageous, owing to its compatibility with offline analysis via MapReduce and real-time analysis via Spark. Three buffer sizes were defined for the receiving end namely, minimum (4096 bytes), default (87,370 bytes), and maximum (4,001,344 bytes). Data transmission was sequential and was determined by the availability of receiving buffer. Figure 4 illustrates the configuration scenario for initial dataset analysis and system performance evaluation.

Data processing evaluation confirmed that the proposed BDA-embedded city architecture has improved data processing time and throughput time, while identifying the threshold values for each city data parameter. Threshold values identified from data analysis are presented in Table 3.

Table 3 indicates the time consumption (*θ*), which represents the summation of time taken for data processing, event generating, and user notifying processes. As revealed from the results, *θ* increases with the dataset size. However, the real-time processing targets streaming data, which are not highly influenced by the dataset size. Nevertheless, a system with high processing speed is essential to handle rapid data generation from smart cities.

### 4.3. Dataset Analysis and Processing Performance Analysis

Smart city realization is highly depending on prompt decision-making, which is supported by real-time BD processing. Autonomous intelligent decision generation supports city municipals to provide high-quality services to the urban community. This section elaborates on dataset analysis, dataset processing performance, and importance of dataset analysis in urban planning.

**Figure 5:**
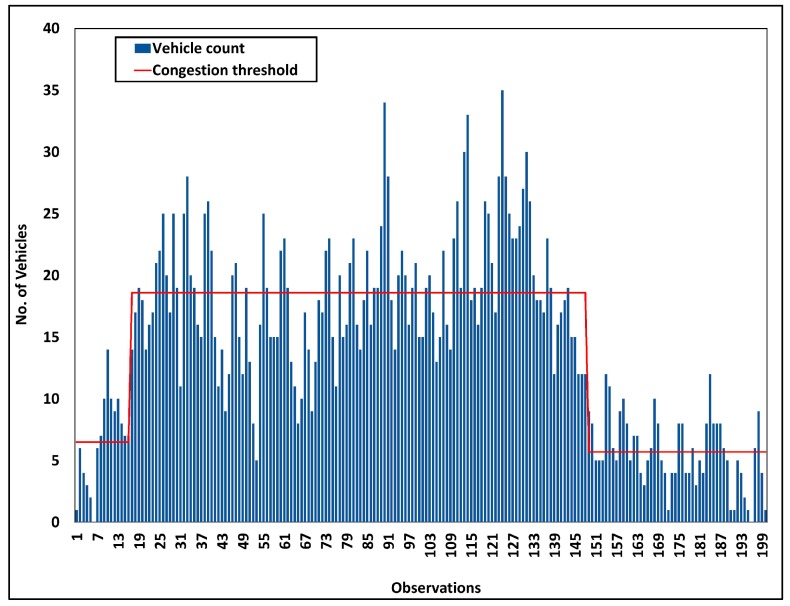
Vehicular density analysis of Aarhus City, Denmark.

**Figure 6:**
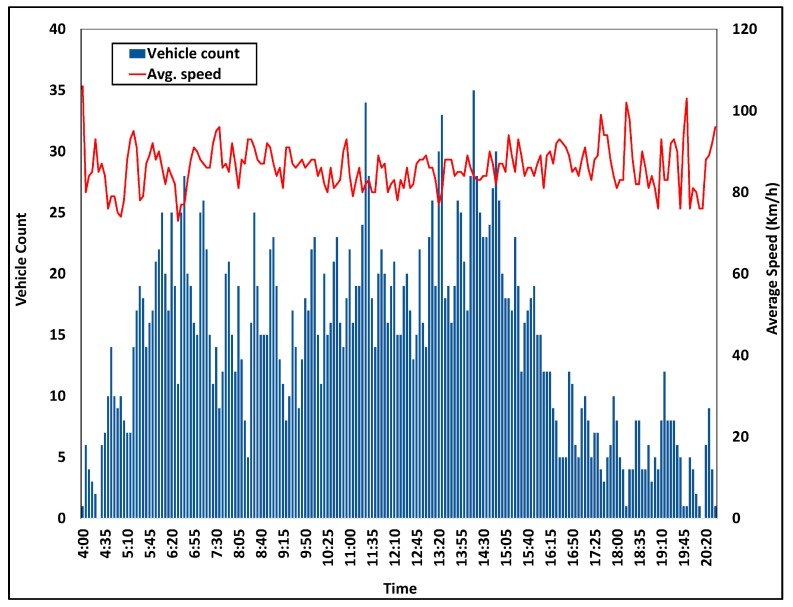
Vehicular speed analysis of Aarhus City, Denmark.

Road traffic data obtained from Aarhus, Denmark has been analyzed to obtain threshold values for vehicular congestion. The dataset contains road traffic data for 3655 m between Aarhus and Hinnerup observation points. The dataset consists of data that were obtained in every 122 s periodically. Road traffic data play a crucial role in smart transportation management. Road congestion status fluctuates with the time of the day. Vehicular dataset analysis results are illustrated in Figure 5 and Figure 6. Figure 5 represents vehicular density variations for each observation. With the aid of proposed data analysis methods, the smart city architecture generates real-time decisions such as traffic congestion updates to drivers. The congestion level threshold varies with time, in order to obtain more realistic decisions. Upon notifying a vehicle count that surpasses the congestion threshold corresponding to that time, the SCSC notifies potential drivers accordingly. Aligning with the analysis results, SCSC is capable of determining road traffic congestion accurately followed by autonomous event generation. Generated events are unicasted from smart transportation components to corresponding service layer components. Subsequently, the traffic management service component broadcasts congestion notifications to all potential drivers and suggest alternative routes to avoid congested road portion.

**Figure 7:**
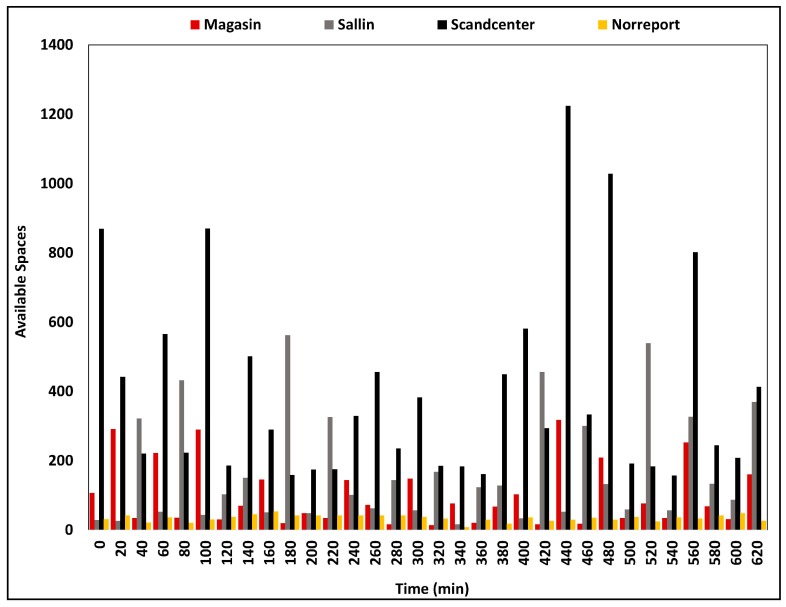
Parking lot availability of Aarhus City, Denmark.

In one hand, parking management is a tedious task for city municipals. On the other hand, finding available parking spaces within city areas is highly challenging for urban citizens. The smart parking component embedded in SCSC is responsible for real-time management of parking lot data and notifying convenient parking spaces to urban citizens. Owing to the benefits of smart parking systems, citizens will be able to find a convenient parking space without any time-consuming observations. The system analyzed Aarhus city’s parking data and updated parking information on Hadoop, via transportation component at SCSC. Citizens can locate available parking spaces by simply accessing the parking management sub-component of smart transportation component. Data collection layer updates parking space availability immediately after being occupied and released. Figure 7 depicts parking availability of different parking lots in Aarhus, Denmark. Parking availability has reduced drastically during the middle of the day. In addition to a parking space locating service, parking management can be improved to serve parking reservation. However, time constraints must be introduced to parking reservation service, to avoid idled parking spaces.

**Figure 8:**
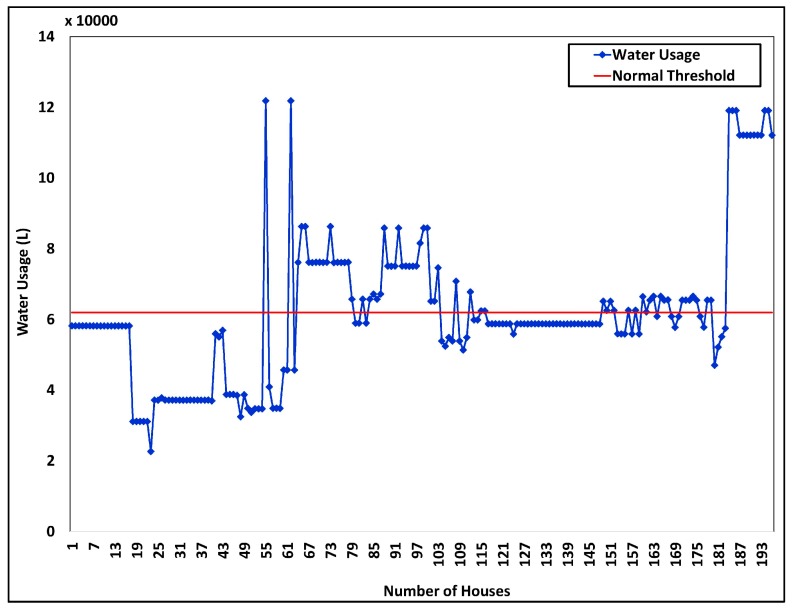
Water consumption analysis of Surrey, Canada.

Urban water consumption has increased alarmingly, due to human activities resulting from drastic urbanization and industrialization. Therefore, the water consumption dataset from Surrey, Canada was analyzed to determine potential solutions for urban water management in future. As shown in Figure 8, in a general household, average monthly water consumption is nearly 60,000 L. This water consumption threshold was obtained by analyzing the water consumption dataset from Surrey, Canada. The intelligent brokers generate warnings to notify users when the water consumption is exceeding the defined threshold value. In addition to controlling residential water consumption, in future municipals can introduce new techniques to reuse water to satisfy demands of the urban community.

**Figure 9:**
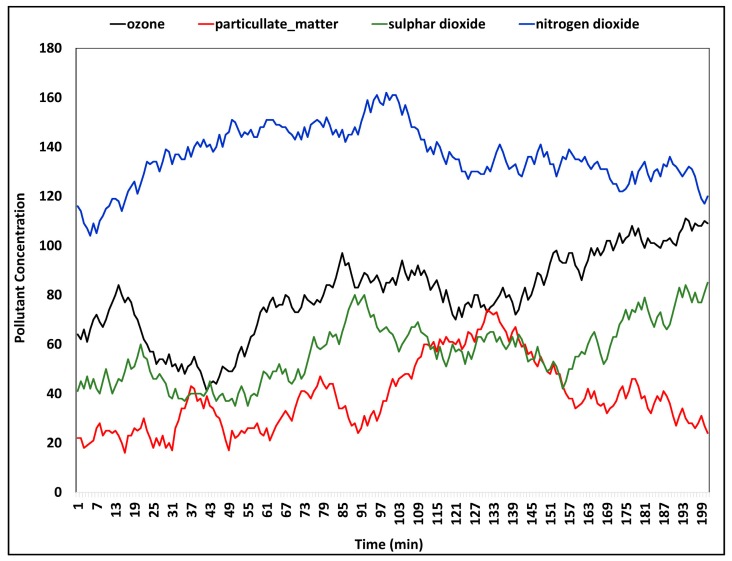
Pollution measurements analysis of Aarhus, Denmark.

Increased waste production, noise pollution, and air pollution result from rapid urbanization cause unfathomable effects for human health as well as the sustainability of environment. With the purpose of determining harmful pollutant concentrations, we analyzed the pollution measurement dataset from Aarhus, Denmark. The analysis was performed on Ozone (O_3_), Nitrogen Dioxide (NO_2_), Sulphur Dioxide (SO_2_), and particulate matter concentration fluctuations during the day are illustrated in Figure 9. Nitrogen dioxide (NO_2_), which results from motor vehicle transportation has the highest concentration. Continuous exposure to NO_2_ causes serious health consequences, such as lung cancers [46]. Increasing concentrations of O_3_ increases greenhouse effect that correlates with global warming. Moreover, O_3_ reactions on skin lipids cause respiratory and skin inflammations [47]. Real-time data analyzing capability of the proposed scheme enable urban citizens to know about pollution levels of city suburbs. Notifying pollution warnings are handled by smart healthcare components and smart meteorology components.

**Figure 10:**
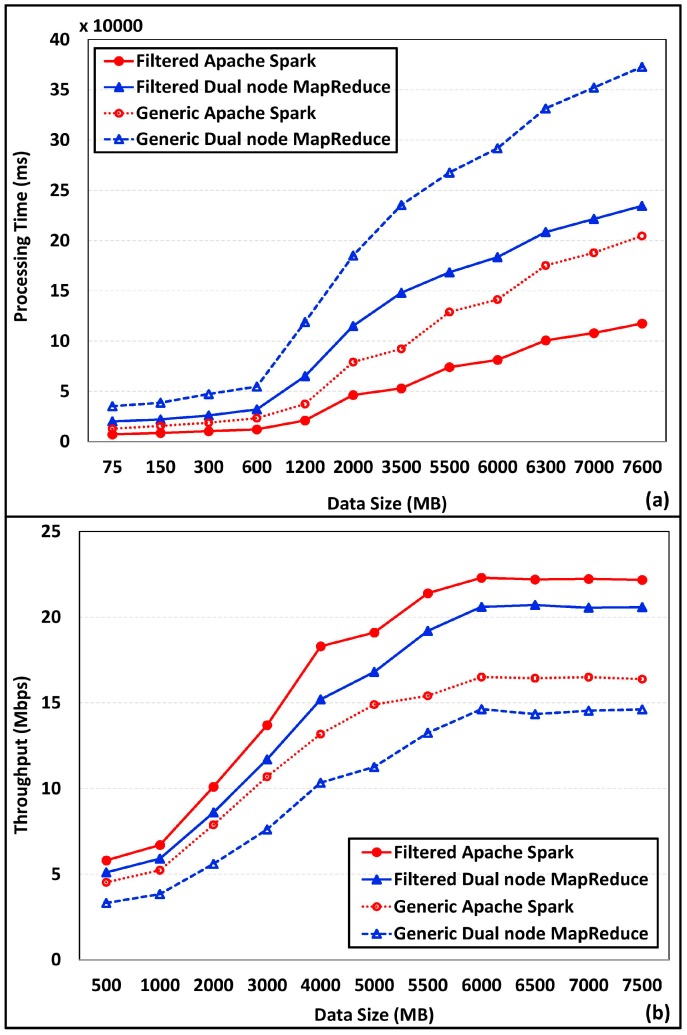
Filtration incorporated processing performance. (**a**) Processing time, (**b**) Throughput.

Data processing time and throughput of the proposed historic and real-time data processing were compared with generic data processing on Spark and dual-node Hadoop MapReduce. Clustered distribution, data filtering, and data normalizing techniques have reduced the processing time, while increasing the throughput. Removal of erroneous and ambiguous data from the analysis datasetin filtration and normalization processes has improved the performance of proposed scheme. Data processing time analysis for offline processing and real-time processing are depicted in Figure 10a. As depicted the processing time for both Spark and MapReduce has been significantly reduced after introducing the filtration techniques. Throughput enhancement gained through the proposed scheme is presented in Figure 10b. Results revealed that the throughput of both Spark and MapReduce increased with the data size.

**Figure 11:**
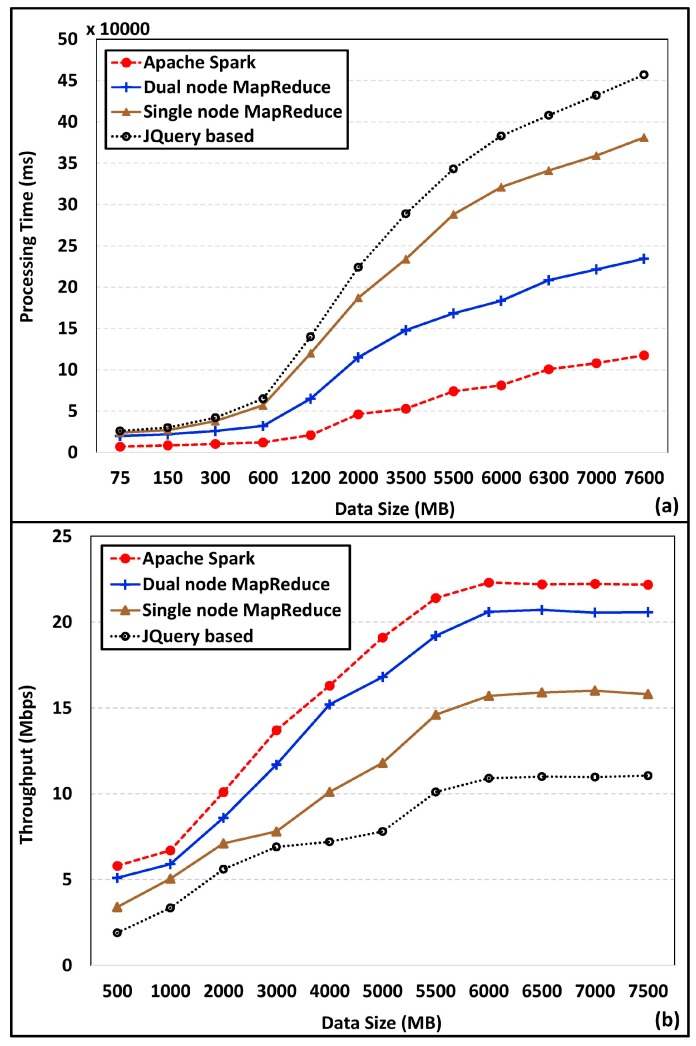
Performance comparison of Spark and MapReduce on dual-node Hadoop. (**a**) Processing time, (**b**) Throughput.

In this work, we occupied MapReduce on a dual-node Hadoop cluster and Apache Spark for offline and real-time data processing respectively. MapReduce on dual-node cluster has improved the processing performance, owing to data distribution. Figure 11a presents data processing time for Apache Spark, MapReduce on dual-node Hadoop, MapReduce on single node Hadoop, and JQuery-based data processing. Processing time increment is rapid with single node Hadoop and JQuery-based processing. Escalated processing time hinders real-time operation of the smart city, due to diminished decision-making capability. Throughput gain of the proposed scheme is compared with MapReduce on single node Hadoop and JQuery-based system, and the results are illustrated in Figure 11b. As shown in Figure 11b, throughput has increased with the data size. Herein we used a fourth generation 3.6 GHz core i5 processor with four cores. Single core applications tend to have a constant throughput, since additional cores do nothing particular and stay idle. A single processor also adheres with a constant throughput, since it cannot distribute its works and it does not support parallelism. However, multicore applications like Hadoop, enforces parallelism by using multiple cores that are available to distribute the load on the processor, while reaching the maximum benefit of available cores [36]. As a result, of parallel distribution, the throughput increase with the data size as well as the core use [36]. With smaller datasets, throughput is less, as the cores are not fully used. However, at one point cores reach to the maximum occupancy level. From that point onwards, the throughput become constant. All four schemes have similar throughput values for small datasets. Degraded data processing performance of single node Hadoop and JQuery system are clearly reflected in the throughput analysis results.

**Figure 12:**
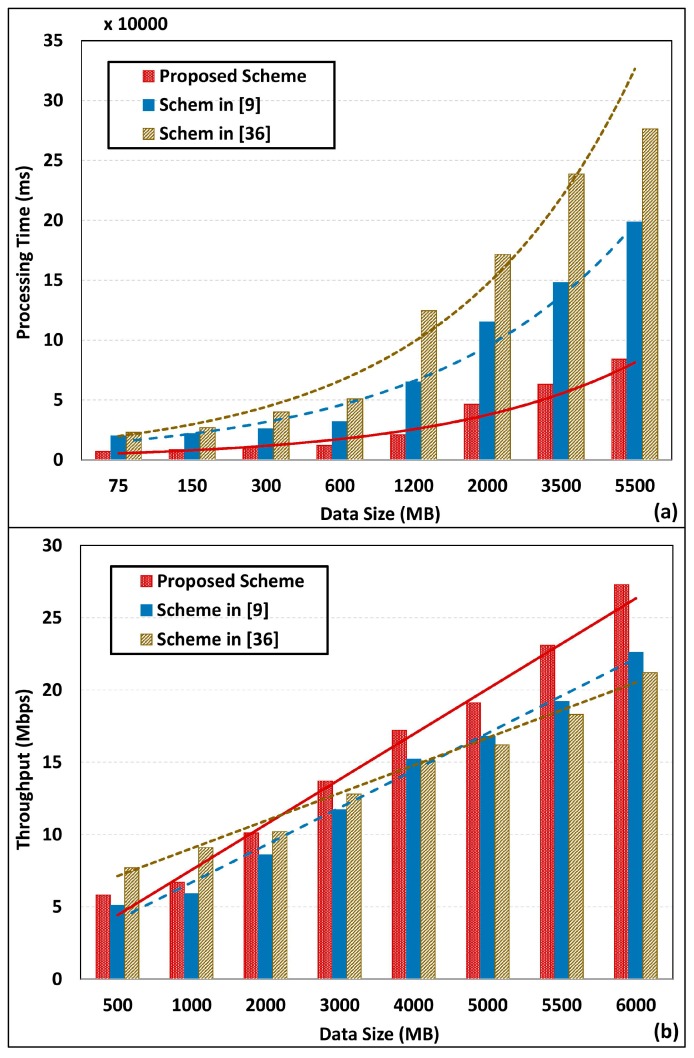
Performance comparison of filtered Spark analysis with existing schemes. (**a**) Processing time analysis with data growth, (**b**) Throughput analysis with data growth.

Figure 12 illustrates the performance analysis of proposed scheme in comparison to one of our previous works [9] and another work proposed by Rathore et.al. [36]. Figure 12a represents processing time analysis with varying dataset sizes up to 5500 MB. As clearly depicted, all three approaches increase the processing time with dataset size. However, the proposed work has obtained the optimal data processing rate compared to works presented in [9] and [36]. As shown in Figure 12b, scheme in [36] has obtained the highest throughput up to 2000 MB. However, the proposed work has taken the lead with datasets that exceed 2000 MB. As clearly illustrated, in comparison, throughput gain with dataset expansion is limited for other schemes, even though the proposed work has achieved a significant throughput gain.

## 5. Conclusions

Smart cities have improved the QoL of urban community with the aid of sophisticated innovations in healthcare, transportation, utility management, and much more, sincea smart city is a consolidation of various smart components. The realization of an intelligent smart city relies on seamless interoperation and coherent integration of all fundamental smart components. In the modern world, smart cities generate data at a very high speed resulting in UBD. Due to exponential data growth, real-time data processing and analysis have become tedious and challenging for smart cities.

Therefore, herein we have proposed a BDA-embedded experimental architecture for smart cities. The BDA-embedded smart city architecture serves two major aspects. Firstly, it facilitates exploitation of UBD in planning, designing, and maintaining smart cities. Secondly, it occupies BDA to manage and process voluminous UBD to enhance the quality of urban services. The architecture consists of three layers that are liable for data aggregation, real-time data management, and service provisioning. Moreover, data normalizing and data filtering techniques are integrated to the proposed work, to further enhance offline and online data processing tasks. Threshold values required for urban planning and city operation management were obtained by analyzing authentic datasets. Performance metrics in terms of online and offline data processing for the proposed dual-node Hadoop cluster is obtained using aforementioned authentic datasets. Throughput and processing time analysis performance were evaluated against existing works, in order to enforce the validity and superiority of the proposed work.. In future, this work can be further improved by incorporating deep-learning algorithms in the decision-making process.

## Figures and Tables

**Table 1:** Smart Meter Data Collection for One Year.

Frequency	1/day	1/hour	1/30 min	1/15 min
Records Collected	365 m	8.75 b	17.52 b	35.04 b
Terabytes Collected	1.82 TB	730 TB	1460 TB	2920 TB

m: million, b: billion.

**Table 2:** Information related to analyzed datasets.

Sources	Dataset	Size
Aarhus City, Denmark [42]	Traffic Data	3.04 GB
Aarhus City, Denmark [43]	Parking lots	0.20 MB
Aarhus City, Denmark [44]	Pollution Data	77.25 MB
Surrey City, Canada [45]	Water Consumption	4 MB

**Table 3:** Event generation performance and threshold limits.

Dataset	Size	Threshold	*θ*
Water Consumption	4 MB	80 Cubic Liters	11.23 s
Traffic Data	3.04 GB	Varies with time	212.88 s
Pollution Data	77.25 MB	80%	16.97 s
Parking lots	0.20 MB	<10/parking garage	3.67 s

## References

[1] Silva B.N., Khan M., Han K. (2017). Internet of Things: A Comprehensive Review of Enabling Technologies, Architecture, and Challenges. IETE Tech. Rev..

[2] Brar G.S., Rani S., Chopra V., Malhotra R., Song H., Ahmed S.H. (2016). Energy efficient direction-based PDORP routing protocol for WSN. IEEE Access.

[3] Jin J., Gubbi J., Marusic S., Palaniswami M. (2014). An information framework for creating a smart city through internet of things. IEEE Internet Things J..

[4] Rani S., Talwar R., Malhotra J., Ahmed S.H., Sarkar M., Song H. (2015). A novel scheme for an energy efficient Internet of Things based on wireless sensor networks. Sensors.

[5] Jung C., Kim K., Seo J., Silva B., Han K. (2017). Topology Configuration and Multihop Routing Protocol for Bluetooth Low Energy Networks. IEEE Access.

[6] Islam S.R., Kwak D., Kabir M.H., Hossain M., Kwak K.-S. (2015). The internet of things for health care: A comprehensive survey. IEEE Access.

[7] Khan M., Silva B.N., Han K. (2016). Internet of Things Based Energy Aware Smart Home Control System. IEEE Access.

[8] Jabbar S., Khan M., Silva B.N., Han K. (2016). A REST-based industrial web of things’ framework for smart warehousing. J. Supercomput..

[9] Silva B.N., Khan M., Han K. (2017). Big Data Analytics Embedded Smart City Architecture for Performance Enhancement through Real-Time Data Processing and Decision-Making. Wirel. Commun. Mob. Comput..

[10] Khan M., Silva B.N., Han K. (2017). A Web of Things-Based Emerging Sensor Network Architecture for Smart Control Systems. Sensors.

[11] Silva B.N., Khan M., Han K. (2018). Load Balancing Integrated Least Slack Time-Based Appliance Scheduling for Smart Home Energy Management. Sensors.

[12] Bouk S.H., Ahmed S.H., Kim D., Song H. (2017). Named-data-networking-based ITS for smart cities. IEEE Commun. Mag..

[13] Siembab W. (1996). Telecity Development Strategy for Sustainable, Livable Communities. The Blue Line Televillage in Compton, California.

[14] Silva B.N., Khan M., Han K. (2018). Towards sustainable smart cities: A review of trends, architectures, components, and open challenges in smart cities. Sustain. Cities Soc..

[15] Alvi A.N., Bouk S.H., Ahmed S.H., Yaqub M.A., Sarkar M., Song H. (2016). BEST-MAC: Bitmap-Assisted Efficient and Scalable TDMA-Based WSN MAC Protocol for Smart Cities. IEEE Access.

[16] Zanella A., Bui N., Castellani A., Vangelista L., Zorzi M. (2014). Internet of things for smart cities. IEEE Internet Things J..

[17] Wenge R., Zhang X., Dave C., Chao L., Hao S. (2014). Smart city architecture: A technology guide for implementation and design challenges. China Commun..

[18] Nandury S.V., Begum B.A. Smart WSN-based ubiquitous architecture for smart cities. Proceedings of the 2015 International Conference on Advances in Computing, Communications and Informatics (ICACCI).

[19] Chen M., Mao S., Liu Y. (2014). Big data: A survey. Mob. Netw. Appl..

[20] Gandomi A., Haider M. (2015). Beyond the hype: Big data concepts, methods, and analytics. Int. J. Inf. Manag..

[21] Fan J., Han F., Liu H. (2014). Challenges of big data analysis. Natl. Sci. Rev..

[22] Cheng B., Longo S., Cirillo F., Bauer M., Kovacs E. Building a big data platform for smart cities: Experience and lessons from santander. Proceedings of the IEEE International Congress on Big Data (BigData Congress).

[23] Silva B.N., Khan M., Han K. (2017). Integration of Big Data analytics embedded smart city architecture with RESTful web of things for efficient service provision and energy management. Futur. Gener. Comput. Syst..

[24] Bouk S.H., Ahmed S.H., Kim D. Vehicular content centric network (VCCN): A survey and research challenges. Proceedings of the 30th Annual ACM Symposium on Applied Computing.

[25] Ahmed S.H., Bouk S.H., Yaqub M.A., Kim D., Song H., Lloret J. (2016). CODIE: Controlled data and interest evaluation in vehicular named data networks. IEEE Trans. Veh. Technol..

[26] Jara A.J., Genoud D., Bocchi Y. Big data in smart cities: From poisson to human dynamics. Proceedings of the 2014 28th International Conference on Advanced Information Networking and Applications Workshops (WAINA).

[27] Sanchez L., Muñoz L., Galache J.A., Sotres P., Santana J.R., Gutierrez V., Ramdhany R., Gluhak A., Krco S., Theodoridis E. (2014). SmartSantander: IoT experimentation over a smart city testbed. Comput. Netw..

[28] Mora-Mora H., Gilart-Iglesias V., Gil D., Sirvent-Llamas A. (2015). A computational architecture based on RFID sensors for traceability in smart cities. Sensors.

[29] Suciu G., Vulpe A., Halunga S., Fratu O., Todoran G., Suciu V. Smart cities built on resilient cloud computing and secure internet of things. Proceedings of the 2013 19th International Conference on Control Systems and Computer Science (CSCS).

[30] Talari S., Shafie-khah M., Siano P., Loia V., Tommasetti A., Catalão J.P. (2017). A review of smart cities based on the internet of things concept. Energies.

[31] Ng S.T., Xu F.J., Yang Y., Lu M. (2017). A master data management solution to unlock the value of big infrastructure data for smart, sustainable and resilient city planning. Procedia Eng..

[32] Mohamed N., Al-Jaroodi J., Jawhar I., Lazarova-Molnar S., Mahmoud S. (2017). SmartCityWare: A service-oriented middleware for cloud and fog enabled smart city services. IEEE Access.

[33] Dunne T. (2012). Big Data, Analytics, and Energy Consumption.

[34] Bestavros A.C.C., Hutyra L., Terzi E. (2016). SCOPE: Smart-city Cloud Based Open Platform and Ecosystem.

[35] FIWARE FIWARE Consolidates as Open Source IoT-enabled Smart Services Platform. https://www.fiware.org/news/fiware-consolidates-as-open-source-iot-enabled-smart-services-platform-of-reference-with-launch-of-fiware-foundation/.

[36] Rathore M.M., Ahmad A., Paul A., Rho S. (2016). Urban planning and building smart cities based on the internet of things using big data analytics. Comput. Netw..

[37] Tang B., Chen Z., Hefferman G., Wei T., He H., Yang Q. A hierarchical distributed fog computing architecture for big data analysis in smart cities. Proceedings of the ASE BigData & SocialInformatics 2015.

[38] Naccarati F., Hobson S. (2011). IBM Smarter City Solutions on Cloud. IBM Global Services White Paper-Government Solutions.

[39] Strohbach M., Ziekow H., Gazis V., Akiva N. (2015). Towards a big data analytics framework for IoT and smart city applications. Modeling and Processing for Next-Generation Big-Data Technologies.

[40] Goldstein M., Uchida S. (2016). A comparative evaluation of unsupervised anomaly detection algorithms for multivariate data. PLoS ONE.

[41] Babar M., Arif F. (2017). Smart urban planning using Big Data analytics to contend with the interoperability in Internet of Things. Futur.Gener. Comput. Syst..

[42] CityPulse, Vehicle Traffic. http://iot.ee.surrey.ac.uk:8080/datasets.html#traffic.

[43] CityPulse, Parking Data Stream. http://iot.ee.surrey.ac.uk:8080/datasets.html#parking.

[44] CityPulse, Pollution Measurements. http://iot.ee.surrey.ac.uk:8080/datasets.html#pollution.

[45] City of Surrey, Water Meters. http://data.surrey.ca/dataset/water-meters/resource/99fe8786-6329-49f7-ae92-2c3b8f6e4778.

[46] Hamra G.B., Laden F., Cohen A.J., Raaschou-Nielsen O., Brauer M., Loomis D. (2015). Lung cancer and exposure to nitrogen dioxide and traffic: A systematic review and meta-analysis. Environ. Health Perspect..

[47] Lakey P.S., Wisthaler A., Berkemeier T., Mikoviny T., Pöschl U., Shiraiwa M. (2017). Chemical kinetics of multiphase reactions between ozone and human skin lipids: Implications for indoor air quality and health effects. Indoor Air.

